# High risk HPV infection prevalence and associated cofactors: a population-based study in female ISSSTE beneficiaries attending the HPV screening and early detection of cervical cancer program

**DOI:** 10.1186/s12885-019-6388-4

**Published:** 2019-12-10

**Authors:** K. Torres-Poveda, I. Ruiz-Fraga, V. Madrid-Marina, M. Chavez, V. Richardson

**Affiliations:** 1 Dirección de Infecciones Crónicas y Cáncer, Centro de Investigación sobre Enfermedades Infecciosas, Instituto Nacional de Salud Pública (INSP), Av. Universidad 655, Santa María Ahuacatitlán, Cuernavaca, 62100 Cuernavaca, Mexico; 2 CONACYT–INSP, Cuernavaca, Morelos Mexico; 3 Subdirección de Prevención y Protección a la Salud, Instituto de Seguridad y Servicios Sociales de los Trabajadores del Estado, (ISSSTE) Cd, Mexico, Mexico

**Keywords:** HR-HPV, HPV screening program, ISSSTE, Mexico

## Abstract

**Background:**

Cervical cancer is the second cause leading of malignancy-related death among Mexican women. The present study determined the population-based prevalence of high risk Human Papillomavirus (HR-HPV) infection and associated cofactors in female beneficiaries of the Institute of Security and Social Services for State Workers (ISSSTE) attending the Program for HPV Screening and Early Detection of Cervical Cancer and registered in the Women’s Cancer Detection System (SIDECAM).

**Methods:**

In a cross-sectional study, cervical samples from 115,651 female users of the program for HPV screening and early detection of cervical cancer recruited in 23 ISSSTE care centers were analyzed for HR-HPV. Logistic regression analyses, adjusting for potential confounders, were performed to determine the association of HR-HPV infection with sexual health and behavior variables and with positivity to cervical premalignant lesions by cytology.

**Results:**

The overall prevalence of HR-HPV infection among female ISSSTE beneficiaries in the 2013–2015 period was 13%. A bivariate analysis of relevant variables for HR-HPV infection showed a statistically significant association for age, number of sexual partners, use of hormonal contraceptives and smoking. A statistical association was found between infection by HR-HPV with the use of hormonal contraceptives, number of sexual partners and smoking and association of HPV 16 and other non-16/18 HR-HPV infection with number of lifetime sexual partners and tobacco use adjusted for age, history of hormonal contraception, number of sexual partners and tobacco use with the exception of exposition variable itself. Similarly, an association was found between HR-HPV infection, regardless of the virus genotype, with positivity to cervical premalignant lesions adjusted for age, number of lifetime sexual partners, history of hormonal contraception and tobacco use.

**Conclusions:**

HR-HPV prevalence in female ISSSTE Women’s Cancer Program users is similar to the population-based prevalence previously reported in Mexican women without cervical alterations. The ISSSTE robust screening and early detection program, based on cytology studies and HPV co-testing, allows us to know the prevalence of HR-HPV infection among female users of the service.

## Background

According to the latest systematic analysis from the Global Burden of Disease Study 2015, 530,000 new cervical cancer (CC) cases were attributable to Human Papillomavirus (HPV) infection [1]. Estimates from the Mexican Burden of Disease Study (MBD-2013) established in 102,241 the number of cancer cases in women, being CC the second most incident neoplasia, only after breast cancer. Age-standardized incidence rate for CC was 12 per 100,000 (12,562 new cases). A ranking of age-standardized mortality rates among Mexican women showed that CC ranked first in those states with higher marginality scores [2].

Worldwide prevalence of HPV infection in women with no cervix abnormalities is 11–12%, with the highest rates being found in sub-Saharan Africa (24%), Eastern Europe (21%), and Latin America (16%) [3]. Age-specific HPV distribution exhibits a peak at young ages (< 25-year-old), and a rebound at older ages (> 45-year-old) in the Americas and Africa. The most prevalent HPV genotypes are HPV-16 (3.2%), HPV-18 (1.4%), HPV-52 (0.9%), HPV-31 (0.8%), and HPV-58 (0.7%) [4]. The prevalence of oncogenic HPV genotypes is increased in women with cervical pathology in proportion to the severity of the premalignant lesion and the burden of HPV infection; thus, cancer is explained by a higher prevalence of potentially oncogenic − 16 and − 18 HPV genotypes [3].

In Mexico, the official standard NOM-014-SSA2–1994 supported by national government covers CC prevention, detection, diagnosis, treatment, control, and epidemiological surveillance. The program for early CC detection consists of a Papanicolaou smear test, along with biomolecular tests for HPV detection as a support for cervical cytology and direct visualization with acetic acid (only when Papanicolaou smear is not available). This detection test should be performed on any asymptomatic women between 25- to 64-year-old or women under 25 and over 64 years of age who show reduced morbidity and the presence of some risk factor associated with CC [5]. The CC Action Program has been implemented to “reduce mortality from cervical cancer in the female population from Mexico”; to achieve this, strategic actions have been taken, including “inter- and intra-sector coordination, timely detection, diagnosis, treatment, quality control, supervision, evaluation and research, and infrastructure strengthening” [6].

About 10% (12 449,609) of the Mexican population is affiliated to the Institute of Security and Social Services for State Workers (ISSSTE); of these, 26.9% (3 346,043) are women in ages from 25 to 64 years, the target group of the Program for HPV Screening and Early Detection of Cervical Cancer and registered in the Women’s Cancer Detection System (SIDECAM); in turn, 1 602,754 ISSSTE affiliates were users of the program in 2013. This study is aimed to assess the prevalence of high-risk- (HR)-HPV infection and associated cofactors among users of the Program for HPV Screening and Early Detection of Cervical Cancer and registered in the SIDECAM.

## Methods

### Study design and population

A cross-sectional study was conducted, with data analysis from a biological data bank of cervical tissue samples built between 2013 and 2015 from 115, 651 female users of the Program for HPV Screening and Early Detection of Cervical Cancer and registered in the SIDECAM attending primary care, reproductive and family planning services in 23 ISSSTE care centers.

**Fig. 1:**
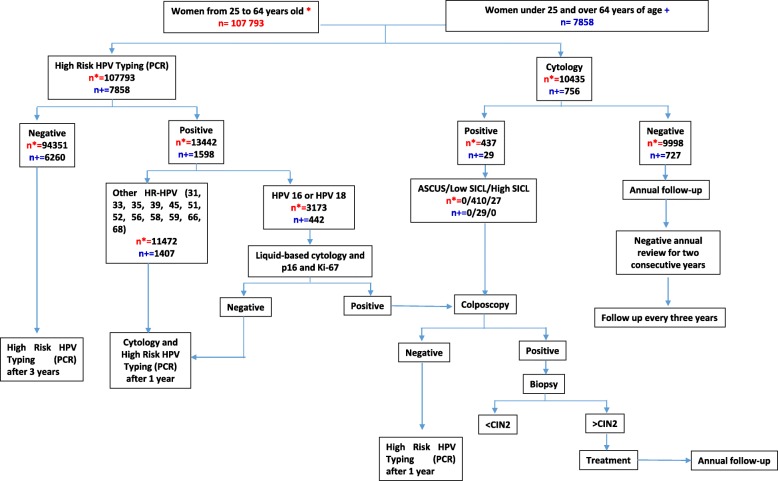
Algorithm for the HPV Screening and Early Detection of Cervical Cancer of the ISSSTE in Mexico. Asymptomatic Women. Women who show reduced morbidity and the presence of some risk factor associated with cervical cancer. Some of the HR-HPV positive cases are positive for both HPV 16 or HPV 18 and other types of HR-HPV. The database used for the analysis only has data of 11,191 cytologies performed and there is no follow-up data of the cases to complete the information on the number of cases in the follow-up points according to the algorithm. SICL: squamous intraepithelial cervical lesion. CIN: cervical intraepitelial neoplasm. ASCUS: atypical squamous cells of undetermined significance. SIDECAM: Women’s Cancer Detection System. ISSSTE: Institute of Security and Social Services for State Workers

The Program for HPV Screening and Early Detection of Cervical Cancer is the population screening program of the ISSSTE aimed at asymptomatic women between 25- to 64-year-old or women under 25 and over 64 years of age who show reduced morbidity and the presence of some risk factor associated with CC. The algorithm that is followed for the HPV screening and early detection of cervical cancer is presented in Fig. 1. For the analysis of this study, we obtained prior authorization for the use of the SIDECAM database of the ISSSTE. As this study was a retrospective confidential analysis of stored databases, no approval from the research and Bioethics Committee was needed.

Sociodemographic, lifestyle, and reproductive data were retrieved from the SIDECAM database for each subject: age, number of lifetime sexual partners, history of consumption of hormonal contraceptives, smoking habit, symptoms referred by the patient, signs observed by colposcopists, PCR-processing laboratory and PCR result for HPV-16, HPV-18, or non-16/18 HR-HPV genotypes.

Information related with the reason for follow-up, result of the last Papanicolaou, diagnosis of premalignant lesion according to Papanicolaou had missing data in the SIDECAM database. The analysis of differential distribution of the reason for follow-up, result of the last Papanicolaou and diagnosis of premalignant lesion between HR-HPV-positive and negative groups was realized in 4176; 11,430 and 11,191 women screened for HR-HPV infection within the Program for HPV Screening and Early Detection of Cervical Cancer and registered in the SIDECAM, respectively.

### Cervical sampling

Healthcare staff assigned to the family planning module of each ISSSTE unit sample endocervical exudates with the PreservCyt vial (Cytyc, Boxborough, MA) for HR-HPV detection and either liquid cytology or Papanicolaou smear. The samples are then sent for analysis to the appropriate ISSSTE pathology unit.

### HR-HPV detection and genotyping in cervical exudate samples

Cervical epithelial scrapings taken from each patient are stored at 4 °C until automated HR-HPV genotyping analysis is done with the Cobas 4800 HPV Test (Roche Molecular Diagnostics, Pleasanton, CA) in the PCR reference laboratory of each ISSSTE regional office. Cobas 4800 is a fully automated real-time PCR system that separately detects HR-HPV-16 and -18 genotypes, as well as ten other HR genotypes (31, 33, 35, 39, 45, 51, 52, 56, 58, 59) and two “probable high risk” strains (66 and 68). HPV testing is performed according to the manufacturer’s directions. For purposes of describing the result of the HR-HPV test in our study, we will describe it as HPV 16 positive, HPV 18 positive and non-16/18 HR-HPV positive.

### Statistical analysis

A bivariate analysis was performed to determine proportion differences of sociodemographic, sexual behavior and obstetric-gynecological history variables, frequency of patient symptoms, and signs reported in cytological studies between groups with and without HR-HPV infection, using the chi-square for categorical variables. Age and the number of sexual partners were recorded as continuous, numeric variables and then analyzed as categorical variables. History of hormonal contraception, smoking habit, patient symptoms and signs according to the cytological study, reason for follow-up, results of last Papanicolaou smear, and diagnosis of premalignant lesion in the population under study were both collected and analyzed as categorical variables. A test for trend across ordered groups (Chi2 test np trend) was used for verifying whether the number of negative and positive for HR-HPV cases followed a linear trend frequency in each group of the analyzed variables.

In the analysis, the subjects were classified as HR-HPV positive when tested positive for any of the 14 HR-HPV types, including HPV-16 and HPV-18. Among these positives, the subjects were subclassified as positive for HPV-16 or HPV-18, regardless of whether they were also positive for another HR-HPV type. The “non-16/18 HR-HPV” group included individuals who were positive for other HR-HPV types (31, 33, 35, 39, 45, 51, 52, 56, 58, 59, 66, and 68), but neither HPV-16 nor HPV-18.

To determine the association between HR-HPV infection, either generic or segregated by genotype, with some risk factor found to be associated in the preliminary bivariate analysis, a logistic regression analysis with odds ratio (OR) calculation and 95% confidence intervals (CI) was performed. Then, a logistic regression analysis was performed adjusting for potential confounders to determine the association of HR-HPV infection, either generic or segregated by genotype, with positivity to cervix premalignant lesion. All statistical analyses were performed with the software STATA v.14.0 (StataCorp, College Station, TX).

## Results

### Prevalence of HR-HPV infection by genotype in the total screened population

The overall prevalence of HR-HPV infection understood as the percentage of women with at least one high-risk HPV genotype detected, in the ISSSTE-affiliated population in the 2013–2015 period was 13%, corresponding to 15,040 beneficiaries. The prevalence of coinfection by HPV 16, HPV 18 and non-16/18 HR-HPV; HPV 16 and HPV 18 only; HPV 16 and non-16/18 HR-HPV and HPV 18 and non-16/18 HR-HPV was 0.06, 0.03, 0.8 and 0.3%, respectively.

The prevalence of single HPV 16 infection was 1.3 and 2.2% with coinfection by other HR-HPV. Single HPV 18 infection prevalence was 0.54 and 0.9% with coinfection with other HR-HPV. The prevalence of single non-16/18 HR-HPV infection and with coinfection with HPV 16 or HPV 18 was 10 and 11%, respectively.

**Fig. 2:**
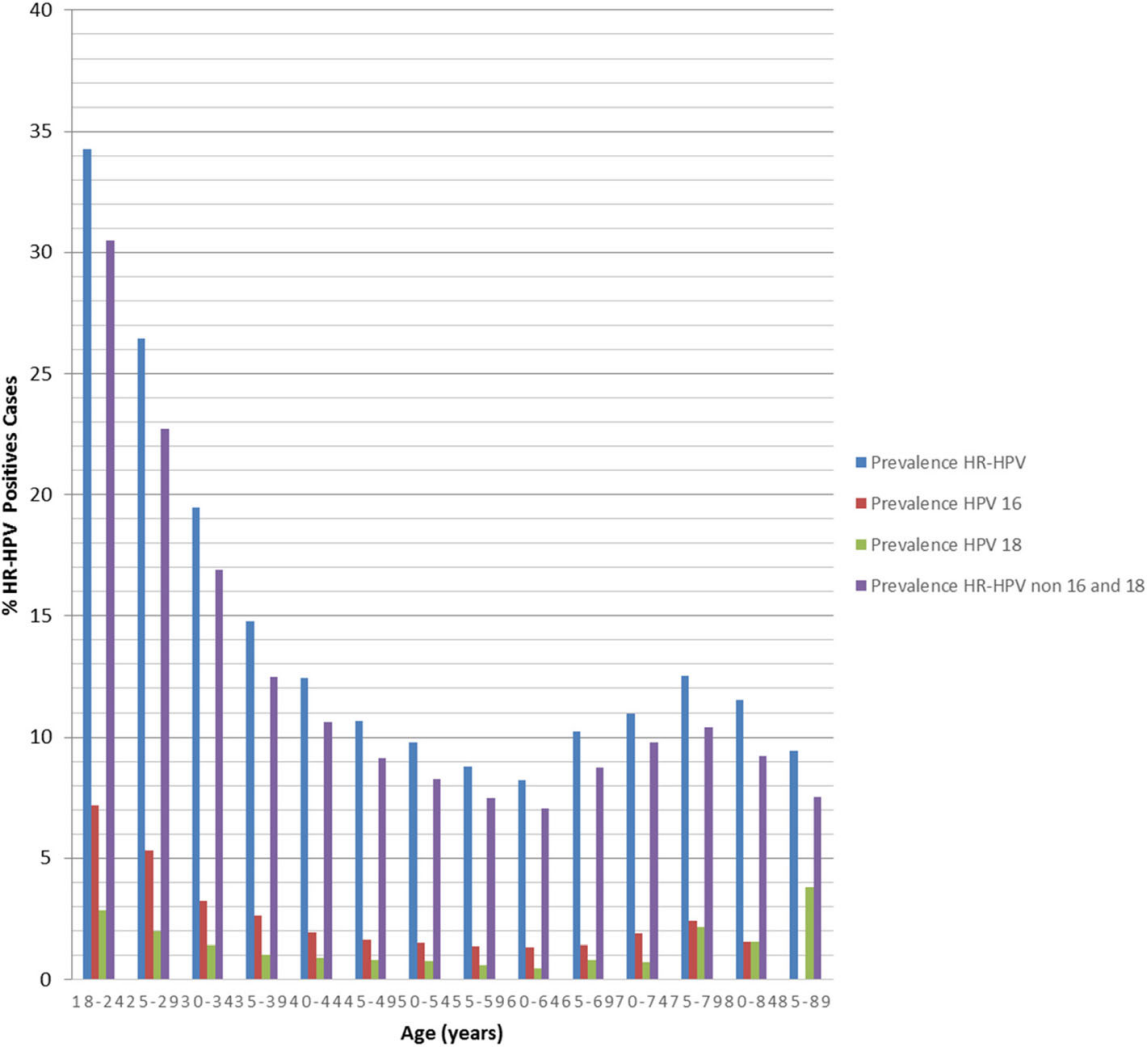
Age-specific prevalence of HR-HPV, HPV16, HPV 18 and/18 non-16/18 HR-HPV. The HR-HPV prevalence understood as the percentage of women with at least one high-risk HPV genotype detected. HPV16, HPV 18 and/18 non-16/18 HR-HPV prevalence understood as the percentage of women with HPV16, HPV 18 and/18 non-16/18 HR-HPV infection (included coinfections with other HR-HPV)

The prevalence of HR-HPV, HPV-16/18 and non-16/18 HR-HPV by age group shows a bimodal distribution with an increased prevalence for the youngest women in the population aged 18–39 with a progressive drop in each age interval and a second bump of positivity for the oldest women aged 65 and above. The prevalence of general HR-HPV, HPV 16 and non-16/18 HR-HPV was higher in the age group of 18 to 24 years (Fig. 2). The mean age of positive cases for HR-HPV infection was 42-year-old, lower than the mean age of negative cases (data not shown).

### Characteristics of the population with HR-HPV

The population under study consisted of 115,651 female subjects for the analysis of HR-HPV infection generic and 114,268 female subjects excluding cases of coinfection with others HR-HPV genotypes for the analysis of HPV-16/18 and non-16/18 HR-HPV. The association analysis of HR-HPV infection, either generic or segregated by genotype, with positivity to cervical premalignant lesion was done with 11,191 subjects. Age distribution in the screened population does not perfectly reflect the target population for the CC early detection program. A higher proportion of subjects in the 18 to 49 years range was found in the screened population with respect to older individuals.

A bivariate analysis of the sociodemographic characteristics, obstetric-gynecological history, and lifestyle-related variables, and lifestyle-related variables and the diagnostic and clinic history of HPV infection of the population screened in the Program for HPV Screening and Early Detection of Cervical Cancer and registered in the SIDECAM is shown in Tables 1 and 2, respectively.

**Table 1:** Characteristics of the population screened for HR-HPV infection in the Program for HPV Screening and Early Detection of Cervical Cancer and registered in the SIDECAM

Characteristics	n (% of Total population screened)	HR-HPV positive n	HR-HPV Prevalence	***np***_***trend***_
Age (years)				**0.0001**
18–24	3238 (2.8)	1109	34	
25–29	5512 (4.7)	1457	26	
30–34	9459 (8.1)	1842	19	
35–39	13,912 (12)	2056	15	
40–44	17,556 (15.1)	2184	12	
45–49	18,859 (16.3)	2012	11	
50–54	20,017 (17.3)	1962	10	
55–59	14,226 (12.3)	1250	9	
60–64	8252 (7.1)	679	8	
65–69	3049 (2.6)	311	10	
70–74	1013 (0.8)	111	11	
75–79	375 (0.3)	47	13	
80–84	130 (0.1)	15	12	
85–89	53 (0.0)	5	9	
Total	115,651	**15,040**	**13**	
Number of lifetime sexual partners				**0.0001**
None	5543	662	12	
1 to 5	106,905	13,606	13	
6 to 10	2585	637	25	
> 11	618	135	22	
History of Hormonal Contraception				**0.0001**
No	101,921	12,985	13	
Yes	13,730	2055	15	
Tobacco Use				**0.0001**
No	101,501	12,813	13	
Yes	14,150	2227	16	

HR-HPV positive n: number of cases with at least one high-risk HPV genotype detected, including cases with co-infection with two or more high-risk HPV genotypes

The HR-HPV prevalence understood as the percentage of women with at least one high-risk HPV genotype detected by specific stratum for each evaluated characteristic

Bold text denotes significant *p* values (p < 0.05). ***np***_***trend***_, Chi2 test np trend to determine proportion differences between groups with and without HR-HPV infection for categorical variables

**Table 2:** Diagnostic and clinic history of HPV infection of the population screened in the Program for HPV Screening and Early Detection of Cervical Cancer and registered in the SIDECAM

Characteristics	n (% of Total population screened)	HR-HPV positive n	HR-HPV Prevalence	***np***_***trend***_
Patient’s symptoms				
Hemorrhage				0.67
No	113,463	14,749	13	
Yes	2188	291	13	
Pruritus				0.36
No	109,159	14,172	13	
Yes	6492	868	13	
Vaginal discharge				**0.0001**
No	92,410	11,514	12	
Yes	23,241	3526	15	
Burning				**0.02**
No	110,398	14,303	13	
Yes	5253	737	14	
Signs observed by colposcopist				
Abnormal cervical neck				0.17
No	110,213	14,300	13	
Yes	5438	740	14	
Ectropion				**0.01**
No	113,947	14,783	13	
Yes	1704	257	15	
Cervicitis				**0.0001**
No	108,564	13,948	13	
Yes	7087	1092	15	
Leucorrhoea				**0.0001**
No	103,850	13,120	13	
Yes	11,801	1920	16	
Abnormal bleeding				**0.01**
No	113,954	14,785	13	
Yes	1697	255	15	
Candidiasis				0.9
No	115,257	14,988	13	
Yes	394	52	13	
Vaginosis				**0.0001**
No	113,279	14,614	13	
Yes	2372	426	18	
Actinomyces				0.18
No	115,599	15,039	13	
Yes	52	10	19	
Trichomonas				
No	115,621	15,031	13	**0.006**
Yes	30	9	30	
Reason for follow-up				**0.0001**
Previous positive HPV result	1979	570	29	
ASCUS o SICL	449	70	16	
Cancer control	1748	199	11	
No information	111,475			
Result of last Papanicolaou				**0.004**
Negative for SICL or malignancy	601	91	15	
Negative with reactive or inflammatory alterations	10,124	1288	13	
Atypical squamous cells of uncertain significance-ASCUS	47	16	34	
SICL low grade CIN 1, condyloma ordinary dysplasia.	439	125	28	
SICL high grade CIN 2, condyloma atypic dysplasia.	22	13	59	
SICL high grade CIN 3 severe dysplasia	3	2	67	
SICL high grade CIN 3, squamous cell carcinoma in situ	2	2	100	
Invasive squamous cell carcinoma	6	1	17	
Atypia of endocervical glandular cells	52	6	12	
Adenocarcinoma In Situ	126	16	13	
No information	104,221			
Diagnosis of SICL				**0.0001**
Negative	10,725	1379	13	
Positive	466	142	30	
No information	104,460			

The HR-HPV prevalence understood as the percentage of women with at least one high-risk HPV genotype detected by specific stratum for each evaluated characteristic

Bold text denotes significant p values (p < 0.05). ***np***_***trend***_, Chi2 test np trend to determine proportion differences between groups with and without HR-HPV infection for categorical variables

SICL: squamous intraepithelial cervical lesion. CIN: cervical intraepitelial neoplasm. ASCUS: atypical squamous cells of undetermined significance. SIDECAM: Women’s Cancer Detection System

Bivariate analysis of relevant lifestyle variables between HR-HPV-positive and negative groups showed a statistically significant association (*p* = 0.0001) for age, number of sexual partners, history of hormonal contraceptives, and smoking habit (Table 1). HR-HPV infection was found in 13% (13,606/106,905) of cases that they referred of 1 to 5 lifetime sexual partners, with a statistically significant trend (np trend = 0.0001) to increasing in the category of 6 to 10 couples (25% - 637/2585) and more 11 couples (22% - 135/618) (Table 1). The prevalence of HR-HPV infection was 15 and 16% in contraceptive and smoking users, respectively.

About the diagnostic and clinic history of HPV infection of the population registered in the SIDECAM, a statistically significant differential distribution of vaginal discharge, burning sensation, cervicitis, leucorrhoea and vaginosis (*p* = 0.0001); ectropion (*p* = 0.01), abnormal bleeding (p = 0.01), and *Trichomonas* sp. infection (*p* = 0.006), was found between HR-HPV-positive and negative groups. The most frequent reason for follow-up in positive HR-HPV cases was a previous HPV diagnosis (68%) and the prevalence of HR-HPV infection was 15% in cases with negative result for SICL in the last Papanicolaou. The prevalence of HR-HPV infection was 30% (142/466) in cases with positive diagnosis of SICL (Table 2).

A bivariate analysis of variables with plausible biological relevance for HR-HPV infection, such as age, number of lifetime sexual partners, history of hormonal contraception, smoking habit and positive leucorrhoea, shows that these factors are related with positivity to any HR-HPV genotype and with positivity to HPV-16 and other non-16/18 HR-HPV (Table 3).

**Table 3:** Characteristics of the population screened for HPV 16, HPV 18 and non-16/18 HR-HPV infection in the Program for HPV Screening and Early Detection of Cervical Cancer and registered in the SIDECAM

	HPV 16 positive n	HPV 16 positive Prevalence	np_trend_	HPV 18 positive n	HPV 18 positive Prevalence	np_trend_	non-16/18 HR-HPV positive n	non-16/18 HR-HPV positive Prevalence	np_trend_
Age (years)			**0.0001**			**0.0001**			**0.0001**
18–24	89	**2.9**		31	1		794	**26**	
25–29	145	2.7		55	1		1069	21	
30–34	172	1.8		66	0.7		1420	15.3	
35–39	228	1.6		83	0.6		1566	11.4	
40–44	225	1.3		93	0.5		1696	9.7	
45–49	187	1		100	0.5		1569	8.3	
50–54	205	1		98	0.4		1525	7.6	
55–59	135	0.9		50	0.3		977	6.9	
60–64	78	0.9		19	0.2		536	6.5	
65–69	25	0.8		20	0.6		244	8.1	
70–74	9	0.9		3	0.3		87	8.7	
75–79	4	1.1		4	1		32	8.7	
80–84	2	1.5		1	0.8		11	8.5	
85–89	0	0		1	2		3	5.8	
Total	1504	**1.3**		624	**0.5**		11,529	**10**	
Number of lifetime sexual partners									
None	60	1.1	**0.0001**	37	0.6	0.30	511	9.3	**0.0001**
1 a 5	1372	1.3		560	0.5		10,428	9.8	
6 a 10	54	2.1		19	0.7		497	19.8	
11 or more	18	3		8	1.3		93	15.4	
History of Hormonal Contraception									
No	1293	1.3	**0.008**	553	0.5	0.72	9963	9.9	**0.0001**
Yes	211	1.6		71	0.5		1566	11.6	
Tobacco Use									
No	1273	1.3	**0.0001**	548	0.5	0.99	9827	9.8	**0.0001**
Yes	231	1.7		76	0.5		1702	12.2	
Ectropion									
No	1478	1.3	0.39	614	0.5	0.77	11,338	11	0.07
Yes	26	1.5		10	0.6		191	11.4	
Leucorrhoea									
No	1319	1.3	**0.005**	546	0.5	**0.05**	10,080	9.8	**0.0001**
Yes	185	1.6		78	0.7		1449	12.5	

The HPV 16, HPV 18 and non-16/18 HR-HPV prevalence results are expressed as percentages of positives women for unique HPV 16, HPV 18 and non-16/18 HR-HPV infections without coinfection with other HR-HPV, by specific stratum for each evaluated characteristic

Bold text denotes significant p values (p < 0.05). ***np***_***trend***_, Chi2 test np trend, to determine proportion differences between positives and negatives groups for HPV 16, HPV 18 and non-16/18 HR-HPV, for categorical variables

A bivariate analysis of relevant variables with biological relevance between groups with and without premalignant cervix lesion according to Papanicolaou smear showed significant differences (*p* = 0.0001) for age, number of lifetime sexual partners, smoking, and molecular HR-HPV diagnosis. No significant differences were observed when comparing diagnosis of cervical premalignant lesions between both groups with respect to the history of hormonal contraception nor with ectropion or leucorrhoea in colposcopic examination (data not shown).

### Association of HR-HPV infection with sexual health, behavioral variables, and risk profile

In addition to age-related risks, the association between HR-HPV infection, either generic or segregated by high risk genotype, and those variables for which biological relevance was determined in the preliminary bivariate analysis, like history of hormonal contraception, number of lifetime sexual partners, and smoking, was evaluated by logistic regression. History of hormonal contraception, number of lifetime sexual partners, and smoking were found to be positively associated with HR-HPV infection adjusting for age, and in the multivariate model (*p* < 0.05). Number of lifetime sexual partners greater than 6 and tobacco use were found associated with HPV 16 infection and other non-16/18 HR-HPV infection in all evaluated models adjusting for age, and in the multivariate model adjusting for age, number of lifetime sexual partners, history of hormonal contraception, and smoking, except for the exposition variable itself. No association was found with HPV-18 infection (Table 4).

**Table 4:** Association between HR-HPV infection and Sexual health and Behavior variables

	HR-HPV n(%)	HR-HPV (by any genotype)		HPV 16 n(%)	HPV 16 infection		HPV 18 n(%)	HPV 18 infection		non-16/18 HR-HPV n(%)	Non-16/18 HR-HPV	
		Age Adjusted OR (95% CI)a	Multivariate Adjusted OR (95% CI)b		Age Adjusted OR (95% CI)a	Multivariate Adjusted OR (95% CI)b		Age Adjusted OR (95% CI)a	Multivariate Adjusted OR (95% CI)b		Age Adjusted OR (95% CI)a	Multivariate Adjusted OR (95% CI)b
History of Hormonal Contraception												
No	12,985(13)	1 (ref)	1 (ref)	2149(2.1)	1 (ref)	1 (ref)	960(0.9)	1 (ref)	1 (ref)	11,115(11)	1 (ref)	1 (ref)
Yes	2055(15)	**1.09(1.03–1.148)**	**1.06(1.008–1.118)**	373(2.7)	1.12(0.968–1.299)	1.09(0.940–1.265)	133(0.9)	0.89(0.700–1.152)	0.90(0.704–1.160)	1764(13)	**1.08(1.027–1.152)**	**1.05(1.003–1.119)**
Number of lifetime sexual partners												
None	662(12)	1 (ref)	1 (ref)	100(1.8)	1 (ref)	1 (ref)	53(0.9)	1 (ref)	1 (ref)	564(10)	1 (ref)	1 (ref)
1 to 5	13,606(13)	1.01(0.937–1.108)	1.00(0.925–1.094)	2292(2.1)	1.14(0.878–1.478)	1.12(0.864–1.455)	984(0.9)	0.76(0.544–1.062)	0.76(0.548–1.071)	11,642(11)	1.01(0.925–1.116)	1.00(0.914–1.103)
6 to 10	637(25)	**1.88(1.665–2.127)**	**1.79(1.590–2.034)**	101(3.9)	**1.60(1.106–2.329)**	**1.51(1.041–2.203)**	42(1.6)	0.95(0.547–1.668)	0.97(0.557–1.711)	564(22)	**1.90(1.665–2.183)**	**1.82(1.590–2.089)**
> 11	135(22)	**1.72(1.395–2.125)**	**1.65(1.336–2.038)**	29(4.7)	**2.39(1.403–4.091)**	**2.27(1.329–3.885)**	14(2.2)	1.77(0.820–3.828)	1.80(0.834–3.906)	109(18)	**1.50(1.185–1.922)**	**1.44(1.137–1.845)**
Tobacco Use												
No	12,813(13)	1 (ref)	1 (ref)	2119(2.1)	1 (ref)	1 (ref)	948(0.9)	1 (ref)	1 (ref)	10,963(11)	1 (ref)	1 (ref)
Yes	2227(16)	**1.21(1.157–1.278)**	**1.18(1.124–1.243)**	403(2.8)	**1.24(1.081–1.435)**	**1.20(1.045–1.392)**	145(1)	0.96(0.755–1.222)	0.95(0.748–1.218)	1916(14)	**1.21(1.147–1.281)**	**1.16(1.102–1.233)**

HR-HPV n(%): number of cases and HR-HPV prevalence understood as the percentage of women with at least one high-risk HPV genotype detected by specific stratum for each evaluated characteristic

HPV 16 n(%): number of cases and prevalence of single HPV 16 infection without coinfection with other HR-HPV, by specific stratum for each evaluated characteristic

HPV 18 n(%): number of cases and prevalence of single HPV 18 infection without coinfection with other HR-HPV, by specific stratum for each evaluated characteristic

Non-16/18 HR-HPV n(%):number of cases and prevalence of single other HR-HPV non 16 and 18 infection without coinfection with HPV 16 or HPV 18, by specific stratum for each evaluated characteristic

a. Odds ratios for all the variables adjusted by age

b. Odds ratios for all the variables adjusted by age, number of lifetime sexual partners, history of hormonal contraception and tobacco use with the exception of exposition variable itself

Statistically significant *p* values ≤0.05 of the OR’s are marked in bold font

An association of HR-HPV infection, regardless of viral genotype, with positivity to premalignant cervix lesion was found in the logistic regression analysis adjusting for age, number of sexual partners, history of hormonal contraceptive and smoking, in agreement with evidence previously published about HR-HPV infection. The association was stronger for HPV-16 infection, followed by non-16/18 HR-HPV infections (Table 5).

**Table 5:** Association between HR-HPV infection and Squamous Intraepithelial Cervical Lesion (SICL)

		HR-HPV infection ***n*** = 15,040		HPV 16 infection ***n*** = 1504		HPV 18 infection ***n*** = 624		Non-16/18 HR-HPV infection ***n*** = 11,529	
		Age Adjusted OR (95% CI)a	Multivariate Adjusted OR (95% CI)b	Age Adjusted OR (95% CI)a	Multivariate Adjusted OR (95% CI)b	Age Adjusted OR (95% CI)a	Multivariate Adjusted OR (95% CI)b	Age Adjusted OR (95% CI)a	Multivariate Adjusted OR (95% CI)b
Diagnosis of SICL									
Negative		1 (ref)	1 (ref)	1 (ref)	1 (ref)	1 (ref)	1 (ref)	1 (ref)	1 (ref)
Positive		**2.55(2.066–3.148)**	**2.51(2.031–3.105)**	**3.48(2.130–5.699)**	**3.36(2.045–5.527)**	1.53(0.607–3.857)	1.38(0.540–3.544)	**2.17(1.72–2.75)**	**2.15(1.70–2.73)**

HR-HPV infection: Positive HR-HPV cases with at least one high-risk HPV genotype detected

HPV 16 infection: Positive cases of single HPV 16 infection without coinfection with other HR-HPV

HPV 18 infection: Positive cases of single HPV 18 infection without coinfection with other HR-HPV

Non-16/18 HR-HPV infection: Positive cases of single other HR-HPV non 16 and 18 infection without coinfection with HPV 16 or HPV 18

a. Odds ratios for all the variables adjusted by age

b. Odds ratios for all the variables adjusted by age, number of lifetime sexual partners, history of hormonal contraception and tobacco use

Statistically significant p values ≤0.05 of the OR’s are marked in bold font

## Discussion

In Mexico, CC prevention has been one of the main objectives in the public health sector for several decades. This led to the development and implementation of the national program of early detection of cancer; in terms of research, this objective also encouraged studies to determine the prevalence of HR-HPV infection. In addition to early CC detection, identifying the prevalence of risk factors involved in HR-HPV infection is relevant, for this would allow the implementation of interventions to prevent and/or treat the disease.

This study is the second largest conducted in Latin America, and the most extensive population-based study to date using the Cobas 4800 system, a highly sensitive and specific test to detect HR-HPV, discriminating HPV-16 and HPV-18 [7]. The overall prevalence of HPV infection in the female ISSSTE-affiliated population in 2013–2015 was 13%, a result comparable with other studies conducted in Mexico. Only two previous studies based on large populations in Mexico have reported HR-HPV specific prevalence estimates. The age distribution of HR-HPV infection in our studied population is similar from those previously reported [8–11].

In the population of our study, the prevalence of HR-HPV shows a first increase at an age of 18, a finding that can be correlated with the sexual initiation in younger women, when they are exposed to HPV for the first time and their adaptive immune response has yet to develop, showing a gradual descent from an age of 25. The maximum peak of HR-HPV prevalence was in ages from 18 to 24 years, with a decrease in older ranges and a marked prevalence increase from the age of 65. Previously, have been reported examples of the U-shaped curve of age-specific HPV prevalence areas in Latin America (Chile, Colombia and Mexico) [12, 13], similar to the findings of our study, regions where a second peak in the oldest group seems to be more prominent [14], could be the result of immune depression, leading to reactivation of previous quiescent infections [15, 16]. However, the second peak in oldest age group could be influenced by the selection criteria inside the program for HPV Screening and Early Detection of Cervical Cancer of women over 64 years of age who show reduced morbidity and the presence of some risk factor associated with CC.

Interestingly, HPV-16/18 prevalence was similar to that reported in a previous study in Mexico [8], but lower than values reported in other populations throughout Latin America; unfortunately, due to the differing designs of these studies, it is difficult to draw conclusions about comparative infection burdens [10, 12, 17, 18]. In addition to the differences in patient-referred symptoms and the signs reported by colposcopists between HR-HPV-positive and negative groups, the variables age, number of sexual partners, history of hormonal contraception, and smoking habit were found to be risk factors for HR-HPV infection, HPV-16 infection, and infection by non-16/18 HR-HPV.

Previous studies found a similar association of a higher number of sexual partners [19–21], history of hormonal contraception [22], and smoking habit [23, 24] with the risk of HR-HPV infection. Higher number of sexual partners as a risk factor for acquiring new HR-HPV infections could be due to changes in the sexual behavior of women and/or their partners, or to a cohort effect [25–27].

Although the association between HR-HPV infection and hormonal contraception found in the multivariate analysis was weak, the proposed association is biologically plausible because the estrogens and progestagens have been reported to interact with hormone receptors, mainly progesterone receptors, in the cervical tissue; additionally, sex steroid hormones are thought to enhance the expression of HPV-16 E6 and E7 oncogenes, promoting the degradation of p53 tumor suppressor genes and enhancing the ability of viral DNA to transform cells [22].

Smoking is another risk factor associated with HR-HPV infection; this could be explained considering that tobacco smoke contains well-known carcinogens that could have a direct transformation effect on cervix tissues and/or cause immunosuppression, allowing HPV infection to persist and progress to cancer [24].

The association herein found between HR-HPV infection and premalignant lesion in the cervix confirms the already known etiological role of HPV in this disease [28–32].

While no cancer records exist to date in Mexico at the national, regional, nor state levels, great efforts have been made within the program for screening and co-testing for CC in the ISSSTE to integrate such screening with an comprehensive care model with a process-oriented approach, going from the traditional cytology-only scheme to a combination of cytology and HR-HPV genotyping, aiming to implement liquid-based cytology in the future, along with HR-HPV genotyping and immunohistochemical tests (p16, ki67). To this end, the ISSSTE has reinforced its infrastructure in terms of cytology laboratory services, molecular biology laboratory, and colposcopy rooms; it also increased its personnel (cytotechnologists, cytopathologists, chemists, and colposcopists) and installed capacity, as reflected in the number of cytology and HR-HPV tests performed per year, to avoid follow-up interruption in women screened as positive.

This study provides information about the prevalence of HR-HPV infection among the female ISSSTE beneficiaries attending in the Program for HPV Screening and Early Detection of Cervical Cancer and registered in the Women’s Cancer Detection System as part of primary care services. It can help policymakers to make better-informed decisions on resource allocation when trying to determine the best screening and triage practices in Mexico.

The main strength of this study is its large-population-based approach. This is the second largest study in Mexico to assess the prevalence of HR-HPV (both HPV-16/18 and non-16/18 HR-HPV). As such, it can also be used in the future to evaluate the impact of HPV vaccination within the ISSSTE female user population. On the other hand, the main limitation of our study is the difficulty to generalize its findings to other populations.

## Conclusion

HR-HPV prevalence in female users of the Program for HPV Screening and Early Detection of Cervical Cancer of the ISSSTE is similar to the population-based prevalence previously reported in Mexican women without cervical alterations. Age, number of sexual partners, history of hormonal contraception, and smoking habit were found to be positively associated with HR-HPV infection; and number of sexual partners and smoking with HPV-16 infection and infection by non-16/18 HR-HPV. The ISSSTE has a robust early detection program based in cytology studies and HPV testing, that allows us to know the prevalence of HR-HPV infection in the female user population.

## Data Availability

The information of the data that supports the findings found in this study, cannot be deposited in publicly available repositories, since they are property of the Institute of Social Security and Services for State Workers (ISSSTE).

## Abbreviations

ASCUS: Atypical squamous cells of undetermined significance
CC: Cervical Cancer
CI: Confidence interval
CIN: Cervical intraepitelial neoplasm
HPV: Human Papillomavirus
HR-HPV: High-risk Human papillomavirus
ISSSTE: Institute of Security and Social Services for State Workers
OR: Odds ratio
PCR: Polymerase chain reaction
SICL: Squamous intraepithelial cervical lesion
SIDECAM: Women’s Cancer Detection System
